# Integrated Prediction System for Individualized Ovarian Stimulation and Ovarian Hyperstimulation Syndrome Prevention: Algorithm Development and Validation

**DOI:** 10.2196/78245

**Published:** 2026-02-03

**Authors:** Jingjing Chen, Jianjuan Zhao, Huiyu Qiu, Yanhui Liu, Yunqi Zhang, Qicheng Sun, Yan Yi, Hongying Tang, Jing Zhao, Bin Xu, Qiong Zhang, Ge Yang, Hui Li, Junjie Liu, Zhongzhou Yang, Shaolin Liang, Yanping Li, Jing Fu

**Affiliations:** 1 Department of Reproductive Medicine Xiangya Hospital Central South University Changsha, Hunan China; 2 Clinical Research Center for Women’s Reproductive Health in Hunan Province Changsha China; 3 Digital Health Lab, Institute for Six-sector Economy Fudan University Shanghai China; 4 Xiangya Hospital Central South University Changsha China; 5 Reproductive Medicine Department The Third Affiliated Hospital of Shenzhen University Shenzhen China; 6 Division of Neonatology Guangzhou Women and Children's Medical Center Guangzhou Medical University Guangdong China; 7 School of Medicine Sun Yat-sen University Guangdong China

**Keywords:** individualized ovarian stimulation, ovarian response, ovarian hyperstimulation syndrome, follicle-stimulating hormone, machine learning

## Abstract

**Background:**

Accurately predicting ovarian response and determining the optimal starting dose of follicle-stimulating hormone (FSH) remain critical yet challenging for effective ovarian stimulation. Currently, there is a lack of a comprehensive model capable of simultaneously forecasting the number of oocytes retrieved (NOR) and assessing the risk of early-onset moderate-to-severe ovarian hyperstimulation syndrome (OHSS).

**Objective:**

This study aimed to establish an integrated mode capable of forecasting the NOR and assessing the risk of early-onset moderate-to-severe OHSS across varying starting doses of FSH.

**Methods:**

This prognostic study included patients undergoing their first ovarian stimulation cycles at 2 independent in vitro fertilization clinics. Automated classifiers were used for variable selection. Machine learning models (11 for NOR and 11 for OHSS) were developed and validated using internal (n=6401) and external (n=3805) datasets. Shapley additive explanation was applied for variable interpretation. The best-performing models were incorporated into a web-based prediction tool.

**Results:**

For NOR prediction, 17 variables were selected, with the gradient boosting regressor achieving the highest performance (internal dataset: *R*^2^=0.7978; external dataset: *R*^2^=0.7924). For OHSS prediction, 19 variables were identified, and the LightGBM model demonstrated superior performance (internal dataset: area under the receiver operating characteristic curve=0.7588; external dataset: area under the receiver operating characteristic curve=0.7287). Shapley additive explanation analysis highlighted the FSH starting dose to BMI ratio and baseline antral follicle count as key predictors for NOR and OHSS, respectively. Dose-response curves were generated to visualize predicted outcomes with varying FSH starting doses. The models were implemented in a user-friendly, research-oriented online prototype, individualized ovarian stimulation guide (InOvaSGuide).

**Conclusions:**

This study introduces an integrated framework for predicting NOR and early-onset moderate-to-severe OHSS risk across different FSH doses. Future prospective evaluation is needed before clinical implementation.

## Introduction

Over the past decade, individualized ovarian stimulation has become a key strategy in in vitro fertilization (IVF). Determining an appropriate starting dose of exogenous follicle-stimulating hormone (FSH) is essential for balancing efficacy and safety. Although earlier clinical practice emphasized maximizing oocyte yield (the more, the better), current consensus favors achieving a moderate ovarian response to optimize live birth rates while minimizing patient discomfort and iatrogenic risks such as ovarian hyperstimulation syndrome (OHSS). Therefore, accurate prediction of ovarian response before stimulation is critical for optimizing treatment outcomes [1-4].

Although biomarkers, including antral follicle count (AFC), anti-Müllerian hormone (AMH) levels, and BMI, are well associated with ovarian response, substantial interindividual and intraindividual variability limits their predictive precision. Tailoring FSH doses based solely on these indicators has not consistently improved clinical outcomes [5,6], highlighting the need for more comprehensive, data-driven approaches that integrate a broader spectrum of clinical and biological factors.

Recent advances in artificial intelligence (AI) and machine learning (ML) offer new opportunities for improving decision-making in assisted reproduction, with applications reported in semen analysis [7], blastocysts grading [8], and trigger-day assessments [9]. Several ML models have also been developed to predict the number of oocytes retrieved (NOR) [10-13] or to classify ovarian responsiveness [10]; however, most remain limited in scope. They typically rely on a narrow set of baseline features, adopt single-model frameworks, and focus predominantly on treatment efficacy such as oocyte yield, with relatively limited attention to safety outcomes, including OHSS. These limitations emphasize the need for predictive frameworks that simultaneously incorporate both efficacy and safety. Furthermore, despite multiple evidence-based algorithms for FSH dosing, considerable variability in ovarian response persists even among patients with comparable baseline characteristics. A model that jointly predicts NOR and OHSS risk across a range of FSH doses may provide useful predictive information and support dose-specific decision-making, helping clinicians consider the balance between efficacy and safety when selecting individualized FSH doses.

In this study, ML models were developed to predict NOR and early-onset moderate-to-severe OHSS using datasets from 2 IVF centers. Models with optimal performance were integrated into a clinician-oriented decision support prototype, termed individualized ovarian stimulation guide (“InOvaSGuide”), complemented by a web-based calculator. For each patient, the system provides individualized dose-response curves that display predicted NOR and early-onset moderate-to-severe OHSS probabilities across varying FSH starting doses, thus supporting personalized ovarian stimulation.

## Methods

### Ethical Considerations

This prognostic study was designed as a retrospective analysis and was approved by the Reproductive Medicine Ethics Committee of Xiangya Hospital (2021010) and the Medicine Ethics Committee of Shenzhen Luohu District People’s Hospital (2024-LHQRMYY-KYLL-63). Informed consent was waived because all data were retrospectively collected from routine clinical records and anonymized before analysis. The study adhered to the Declaration of Helsinki and followed the TRIPOD (Transparent Reporting of a Multivariable Prediction Model for Individual Prognosis or Diagnosis; Table S1 in Multimedia Appendix 1) reporting guideline [14]. All data analyses were performed by an external team using anonymized data only, ensuring full protection of participant privacy. No compensation was provided to participants, as the study involved retrospective and fully anonymized data.

### Study Cohort

The inclusion criteria were (1) patients with the first ovarian stimulation cycle conducted between January 1, 2018, and September 30, 2022, at the Department of Reproductive Medicine of Xiangya Hospital (internal dataset) and between May 1, 2021, and December 30, 2023, at the Reproductive Center of Shenzhen Luohu District People’s Hospital (external dataset) and (2) patients aged 20 to 40 years. Exclusion criteria were (1) patients with diminished ovarian reserve, diagnosed by AMH ≤1.1 ng/mL or baseline AFC ≤7 [15]; (2) patients using the microstimulation protocols for ovarian stimulation, including progestin-primed ovarian stimulation protocol, natural cycle protocol, etc; and (3) patients with more than 50% missingness in key clinical variables. Notably, patients with diminished ovarian reserve or those undergoing microstimulation protocols were excluded because these groups require highly individualized stimulation strategies, exhibit markedly lower oocyte yield, and have a substantially reduced risk of OHSS under comparable FSH exposure, which would have created pronounced class imbalance and reduced model robustness.

After screening, 6401 patients from Xiangya Hospital and 3805 from Shenzhen Luohu District People’s Hospital were included in the internal and external datasets, respectively.

### Ovarian Stimulation Process and the Diagnosis of Early-Onset Moderate-to-Severe OHSS

Before commencing the IVF and intracytoplasmic sperm injection cycle, patients underwent a thorough physical examination, including the assessment of basic physical parameters (height, weight, and BMI), measurement of basal hormone levels (FSH, luteinizing hormone [LH], estradiol, testosterone, progesterone, prolactin, and AMH), biochemical tests (fasting glucose, fasting insulin, lipid levels, and thyroid hormones), and transvaginal ultrasonography for basal AFC. Subsequently, experienced physicians personalized the ovarian stimulation protocol and the starting dose of FSH based on comprehensive clinical assessment. Throughout stimulation, patients underwent monitoring via transvaginal ultrasonography and serum hormone assessments, with gonadotropin dosage adjustments made according to individual ovarian responses. Human chorionic gonadotropin or gonadotropin-releasing hormone agonist, alone or combined, triggered oocyte maturation when 3 or more follicles measuring 17 mm or greater were observed. Oocyte retrieval occurred 36 hours after triggering, and the NOR was recorded. Eligible patients underwent fresh embryo transfer with 1 or 2 embryos.

The study primarily focused on the occurrence of early-onset, moderate-to-severe OHSS, which was diagnosed within 9 days after triggering based on established guidelines [16], considering both clinical and laboratory features. All relevant individual and clinical variables during the process were obtained from the clinical database for feature screening and selection.

### Data Preprocessing and Feature Selection

All data analysis was performed by an external team using anonymized data, ensuring full protection of participant privacy. To elucidate correlations between predictive features and clinical outcomes with an emphasis on medical interpretability, we performed feature engineering on selected variables. This process resulted in 2 additional variables: “FSH to LH ratio” and “FSH starting dose to BMI ratio,” which improved predictive accuracy while maintaining transparency and clinical relevance. Furthermore, to address skewness and improve distributional normality, a logarithmic transformation was applied to AMH, triglycerides, and FSH/LH (Figures S1 and S2 in Multimedia Appendix 1). Missing data were handled using mean imputation, with feature-wise means computed exclusively from the training set and subsequently applied to the test and external validation sets, to prevent information leakage. The overall proportion of missingness was low, and imputation did not materially alter variable distributions.

To identify key variables, we applied feature importance–based selection using the Boruta algorithm, performed exclusively within the training dataset (Figures S3 and S4 in Multimedia Appendix 1). This approach led to the selection of 17 variables for the NOR prediction model and 19 variables for the OHSS prediction model.

### NOR Model Development

In the prediction of NOR, 17 features, including starting dose of FSH to BMI ratio, BMI, log (AMH), and specifically the ovarian stimulation protocol, were selected. The NOR divided by the starting dose of FSH was used and logarithmically transformed as the outcome variable with improved predictive performance. For model training, the internal dataset was divided into an 8:2 split, with 79.9% (5120/6401) of the data randomly allocated to the training set and the remaining 20% (1281/6401) assigned to the internal test set. All data from the external dataset were held out entirely and used exclusively as an external validation cohort, providing an independent assessment of model generalizability across institutions. Eleven ML algorithms, including a linear regression model, were trained to predict the preprocessed NOR outcome. Hyperparameter tuning was performed using 5-fold cross-validation within the training set only, with all hyperparameters predefined and summarized in Table S2 in Multimedia Appendix 1. Model performance was assessed using 4 key metrics: *R*^2^, adjusted *R*^2^, mean absolute error, and root mean square error.

### OHSS Model Development

For OHSS prediction, the target variable was the occurrence of early-onset moderate-to-severe OHSS. Given the low event rate and resulting class imbalance, several commonly used imbalances handling strategies (eg, oversampling, undersampling, and ensemble-based resampling) were evaluated. Cost-sensitive learning was ultimately adopted, assigning differentiated penalties to misclassifications while preserving all original clinical data distribution. To prevent overfitting, model complexity was controlled by limiting the number of parameters and applying regularization techniques, as appropriate for each algorithm. Eleven ML algorithms were implemented, with corresponding hyperparameters detailed in Table S3 in Multimedia Appendix 1. As with the NOR model, hyperparameter optimization was conducted exclusively within the training set, and model performance was evaluated on both the internal and the external datasets using the area under the receiver operating characteristic curve (ROC-AUC), the precision-recall area under the curve (PR-AUC), recall, specificity, weighted *F*_1_-score, Cohen κ, and positive and negative predictive values.

### Shapley Additive Explanation Value

To further explore the significant features driving the model’s predictions, we used the Shapley additive explanation (SHAP) analysis to assess the importance of core features. SHAP serves as an interpretative tool for ensemble tree models, offering a detailed breakdown of the influence of input features on predictions.

### Creation of Dose-Response Curves

In this study, 2 distinct models were developed: a classification model for predicting early-onset moderate-to-severe OHSS and a regression model for forecasting NOR. Models of best performance were incorporated into an integrated, research-oriented computational system, complemented by a web-based calculator. By inputting baseline patient characteristics, the system generates predictions for NOR and early-onset moderate-to-severe OHSS probability, presented as a dose-response curve illustrating changes with increasing FSH starting doses.

### Statistical Analysis

The baseline characteristics of patients between the internal dataset and the external dataset were compared using the chi-square test for categorical variables. For continuous variables, we assessed normality using the Shapiro-Wilk W test. Depending on the results, we used either the 2-tailed Student *t* test or the Mann-Whitney *U* test for comparison. R Studio (version 4.3.1; R Foundation for Statistical Computing), Python (version 3.11.4; Python Software Foundation), the open-source *scikit-learn* package (version 3.9.13; open-source community-developed Python ML library), LightGBM (version 4.3.0; Microsoft Corporation), and XGBoost (version 2.0.0; open-source project maintained by XGBoost contributors) were used for model development and statistical analyses.

## Results

### The Integrated Ovarian Response Prediction System

**Figure 1:**
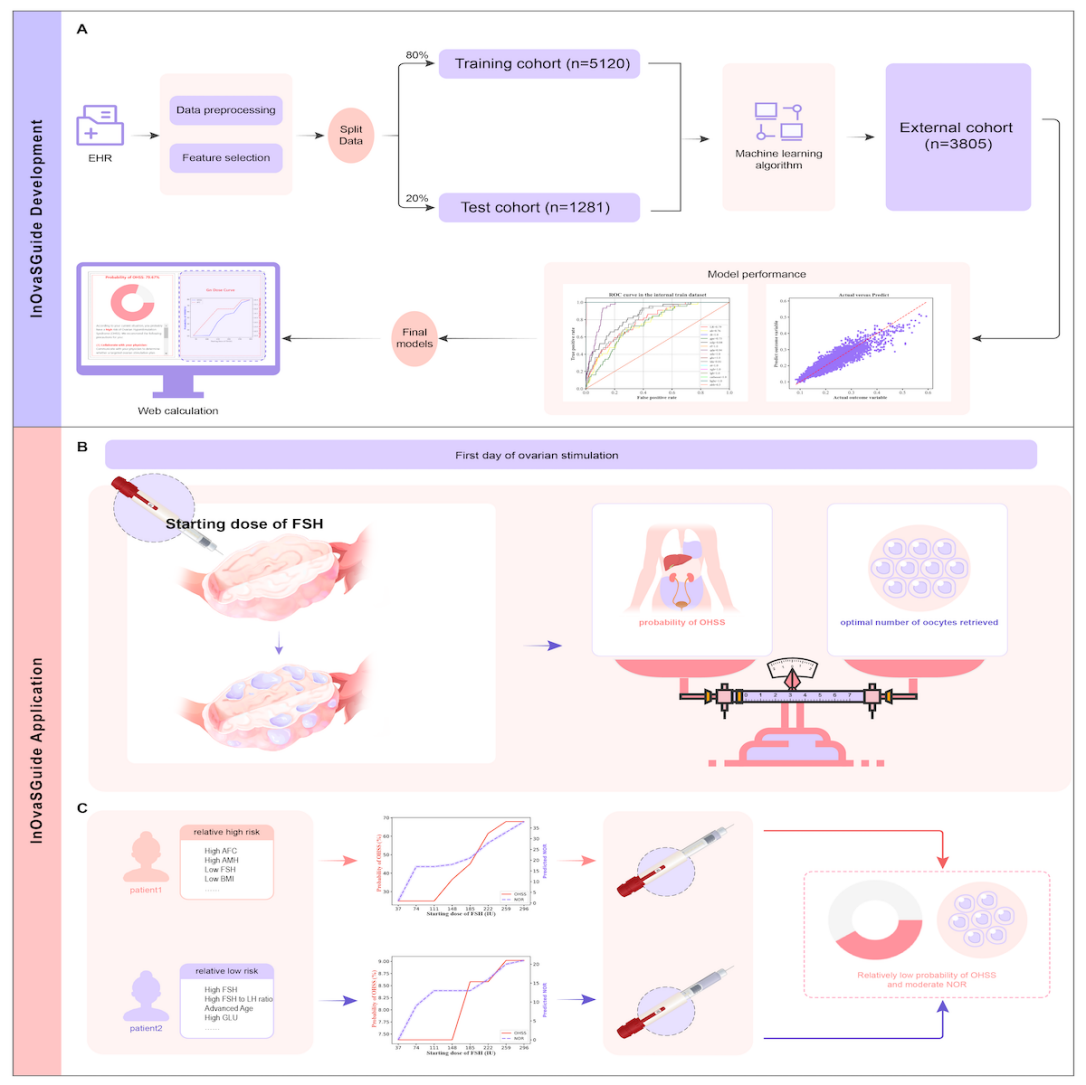
Flowchart of the study: (A) the modeling process with internal and external datasets, (B) illustration of the primary goal of this study, and (C) illustration of the clinical application of the individualized ovarian stimulation guide (InOvaSGuide) system. EHR: electronic health record; FSH: follicle-stimulating hormone; NOR: number of oocytes retrieved; OHSS: ovarian hyperstimulation syndrome.

To address the challenges of individualized ovarian stimulation, we developed an integrated prediction system, “InOvaSGuide,” designed to predict both the NOR and the probability of early-onset moderate-to-severe OHSS before ovarian stimulation. The system was built using datasets from 2 IVF clinics and incorporates 2 distinct ML models for NOR and OHSS predictions, respectively (Figure 1A). By analyzing patients’ baseline characteristics, the system generates dose-response curves that illustrate the predicted benefit (NOR) and risk (early-onset moderate-to-severe OHSS) across varying FSH starting doses (Figures 1B and C). Additionally, a user-friendly web-based calculator was developed to enhance accessibility and support exploratory use in clinically relevant contexts (Figure S7 in Multimedia Appendix 1).

### Patient Characteristics

A total of 6401 patients from the internal dataset and 3805 patients from the external dataset were included, with baseline characteristics detailed in Table 1. The median age was 30.0 (IQR 27.0-33.0) years in the internal dataset and 32.0 (IQR 29.0-35.0) years in the external dataset. The gonadotropin-releasing hormone antagonist protocol was the most commonly used in both datasets (internal dataset: 2650/6401, 41.4%; external dataset: 1913/3805, 50.3%). The median number of NOR was 13.0 (IQR 9.0-17.0) and 15.0 (IQR 10.0-20.0) for the internal and external dataset, respectively. In the internal dataset, 55 (0.9%) patients were diagnosed with moderate-to-severe OHSS, whereas 46 (1.2%) patients were diagnosed in the external dataset. Further comparisons between OHSS and non-OHSS cases in both datasets are detailed in Tables S4 and S5 in Multimedia Appendix 1.

**Table 1:** Baseline characteristics of the patients in the internal and external datasets.

			Internal dataset (n=6401)		External dataset (n=3805)		*P* value	
Age (y), median (IQR)			30.0 (27.0-33.0)		32.0 (29.0-35.0)		<.001	
BMI (kg/m^2^), median (IQR)			21.7 (19.8-24.0)		21.6 (19.8-23.6)		.02	
Baseline FSH^a^ (mIU/mL), median (IQR)			6.2 (5.2-7.2)		6.5 (5.5-7.5)		<.001	
Baseline luteinizing hormone (mIU/mL), median (IQR)			5.3 (3.8-7.1)		5.2 (3.7-6.9)		.006	
Anti-Müllerian hormone (ng/mL), median (IQR)			4.1 (2.6-5.4)		3.8 (2.5-5.7)		.20	
Fasting blood glucose (mmol/L), median (IQR)			5.3 (5.1-5.4)		4.5 (4.3-4.8)		<.001	
Fasting insulin (μU/mL), median (IQR)			11.0 (7.5-12.1)		62.6 (62.6-62.6)		<.001	
Homeostasis model assessment of insulin resistance, median (IQR)			2.5 (1.7-2.9)		13.3 (13.3-13.3)		<.001	
Baseline antral follicle count, median (IQR)			20.0 (14.0-24.0)		13.0 (10.0-19.0)		<.001	
**Ovarian stimulation protocol, n (%)**								<.001
	GnRH^b^ agonist long protocol	1211 (18.9)		0 (0)				
	GnRH antagonist protocol	2650 (41.4)		1913 (50.3)				
	Early-follicular phase long-acting GnRH agonist long protocol	2300 (35.9)		1827 (48)				
	Ultralong GnRH agonist protocol	240 (3.8)		65 (1.7)				
Starting dose of FSH (IU), median (IQR)			150.0 (150.0-187.5)		225.0 (150.0-300.0)		<.001	
Total dose of FSH (IU), median (IQR)			1950.0 (1500.0-2437.5)		2100.0 (1575.0-2750.0)		<.001	
Estradiol level on the day of triggering (pg/mL), median (IQR)			3269.3 (2580.0-3269.3)		2750.0 (1804.0-3961.0)		<.001	
Oocytes retrieved, median (IQR)			13.0 (9.0-17.0)		15.0 (10.0-20.0)		<.001	
**Degree of ovarian hyperstimulation syndrome, n (%)**								.11
	Normal	6346 (99.1)		3759 (98.8)				
	Moderate to severe	55 (0.9)		46 (1.2)				

^a^FSH: follicle-stimulating hormone.

^b^GnRH: gonadotropin-releasing hormone.

### Model Performance

**Figure 2:**
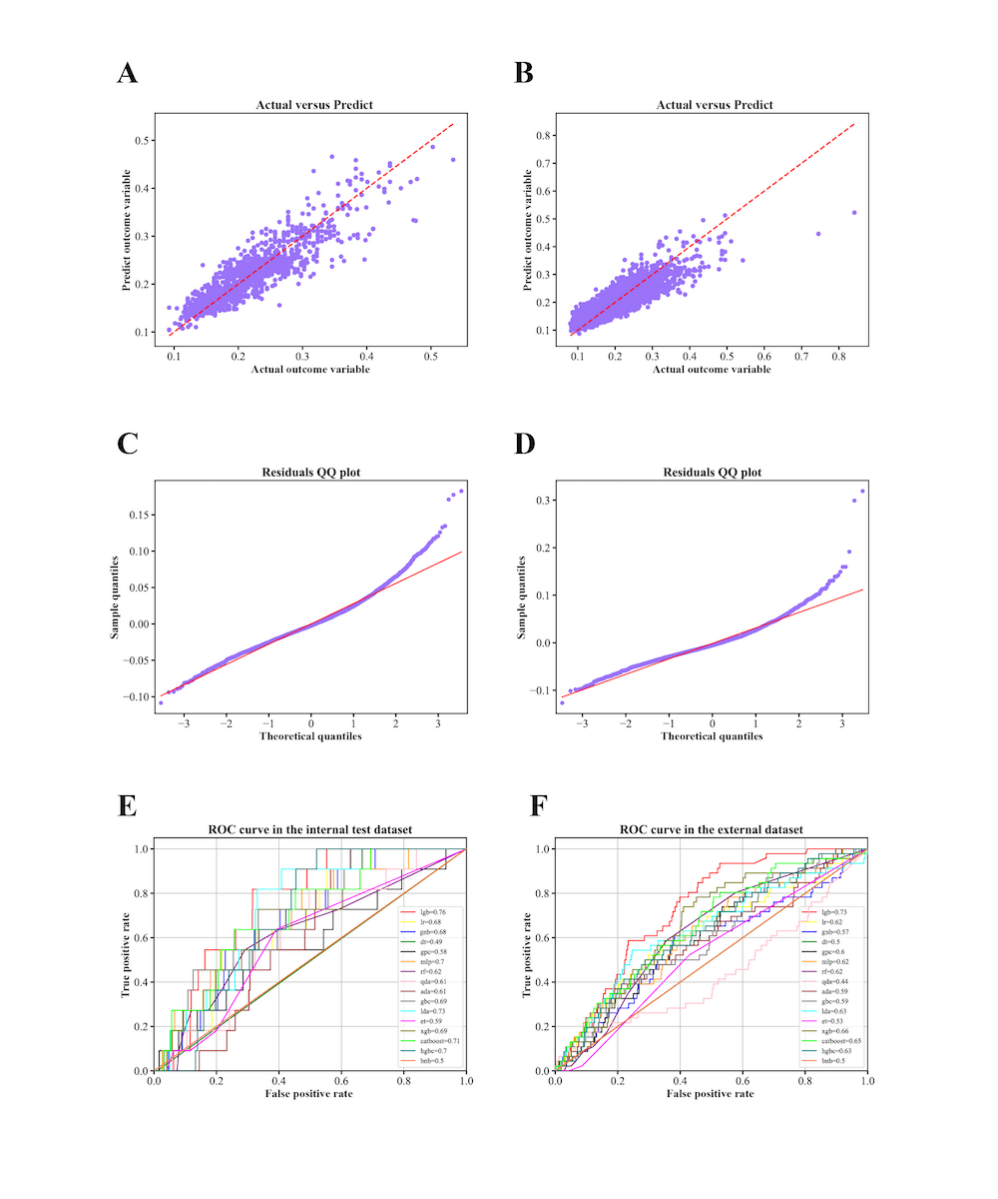
Model performance in predicting the number of oocytes retrieved (NOR; A–D) and ovarian hyperstimulation syndrome (OHSS; E and F) in the internal and external datasets. ADA: adaptive boost classifier; BNB: Bernoulli Naive Bayes; CatBoost: categorical boosting classifier; DT: decision tree classifier; ET: extra trees classifier; GBC: gradient boosting classifier; GNB: Gaussian Naive Bayes; GPC: Gaussian process classifier; HGBC: histogram-based gradient boosting classifier; LDA: linear discriminant analysis; LGB: light gradient boosting machine classifier; LR: logistic regression; MLP: multilayer perceptron classifier; QDA: quadratic discriminant analysis; QQ: quantile-quantile; RF: random forest; ROC: receiver operating characteristic; XGB: extreme gradient boosting classifier.

For NOR prediction, the gradient boosting regressor exhibited the best performance, with an *R*^2^ value of 0.7978 in the internal dataset and 0.7924 in the external dataset, indicating strong explanatory power (Table 2). The model’s mean absolute error was 0.0223 and the root mean square error was 0.0298, collectively affirming the high accuracy and minimal bias. The model’s predictions aligned closely with the actual outcomes, demonstrating relatively high accuracy. The Quantile-Quantile plot further confirmed that the residuals followed a normal distribution, as they closely aligned with the diagonal line (Figures 2A-2D).

**Table 2:** Performance metrics of the number of oocytes retrieved prediction models in internal and external datasets.

Machine learning models	Internal dataset					External dataset			
	*R* ^2^	Adjusted *R*^2^	Mean absolute error	Root mean square error	*R* ^2^		Adjusted *R*^2^	Mean absolute error	Root mean square error
Gradient boosting regressor	0.7978	0.7951	0.0223	0.0298	0.7924		0.7915	0.0243	0.0327
Light gradient boosting machine regressor	0.7908	0.7880	0.0228	0.0303	0.7826		0.7817	0.0243	0.0334
Extreme gradient boosting regressor	0.7889	0.7861	0.0228	0.0305	0.7907		0.7898	0.0236	0.0328
Random forest regressor	0.7849	0.7820	0.0229	0.0308	0.8020		0.8012	0.0229	0.0319
Ridge	0.7463	0.7429	0.0253	0.0334	0.3228		0.3198	0.0470	0.0590
Linear regression	0.7463	0.7428	0.0253	0.0334	0.3235		0.3204	0.0469	0.0590
Decision tree regressor	0.5452	0.5391	0.0330	0.0447	0.6108		0.6090	0.0318	0.0447
Support vector regression	0.5107	0.5041	0.0390	0.0464	0.5321		0.5300	0.0366	0.0490
Lasso	−0.0023	−0.0158	0.0519	0.0664	−0.1773		−0.1826	0.0648	0.0778
Elastic net	−0.0023	−0.0158	0.0519	0.0664	−0.1773		−0.1826	0.0648	0.0778
Multilayer perceptron regressor	−0.4608	−0.4805	0.0523	0.0802	−351.3296		−352.9112	0.6948	1.3453

For early-onset moderate-to-severe OHSS prediction, the LightGBM model consistently outperformed other algorithms, achieving an ROC-AUC of 0.7588 in the internal dataset and 0.7287 in the external dataset (Figures 2E and 2F). While recall, specificity, weighted *F*_1_-score, and Cohen κ score indicated reasonable discriminative performance, precision-related metrics, including positive predictive value, negative predictive value, and PR-AUC, remained modest across all classifiers. The results, along with the confusion matrices, are summarized in Table 3 and Figures S5 and S6 in Multimedia Appendix 1.

**Table 3:** Performance metrics of early-onset moderate-to-severe ovarian hyperstimulation syndrome prediction models in internal and external datasets.

Classifier	Internal dataset									External dataset							
	ROC-AUC^a^	Recall	Specificity	Weighted *F*_1_-score	κ	PPV^b^	NPV^c^	Precision-recall AUC^d^	ROC-AUC		Recall	Specificity	Weighted *F*_1_-score	κ	PPV	NPV	Precision-recall-AUC
LGBMClassifier^e^	0.7588	1.0000	0.9833	0.3466	0.5044	0.3409	1.0000	0.0176	0.7287		0.9348	0.9859	0.4523	0.6099	0.4464	0.9982	0.0227
LinearDiscriminantAnalysis	0.7313	1.0000	0.5690	0.0281	0.0384	0.0197	1.0000	0.0162	0.6574		0.6739	0.9560	0.5966	0.7362	0.5956	0.9934	0.0196
CatBoost^f^	0.7059	1.0000	0.9584	0.1795	0.2918	0.1724	1.0000	0.0173	0.6527		0.8043	0.9852	0.4045	0.5635	0.3996	0.9940	0.0206
MLPClassifier^g^	0.6966	0.9091	0.9784	0.2724	0.4177	0.2669	0.9971	0.0168	0.6322		0.8913	0.9996	0.2071	0.3275	0.1987	0.9893	0.0236
GradientBoostingClassifier	0.6930	1.0000	0.9610	0.1889	0.3053	0.1819	1.0000	0.0161	0.6235		0.2174	0.9553	0.8757	0.9227	0.8837	0.9947	0.0157
XGBClassifier^h^	0.6892	0.7273	0.9941	0.5199	0.6759	0.5181	0.9955	0.0152	0.6221		0.8043	0.9944	0.4263	0.5854	0.4217	0.9933	0.0189
GaussianNB^i^	0.6808	1.0000	0.9755	0.2678	0.4111	0.2614	1.0000	0.0135	0.6186		0.7609	0.9999	0.3548	0.5113	0.3498	0.9883	0.0276
LogisticRegression	0.6791	1.0000	0.4837	0.0250	0.0324	0.0165	1.0000	0.0138	0.6019		1.0000	0.9827	0.0121	0.0003	0.0000	0.9919	0.0182
RandomForest	0.6178	0.7273	0.9909	0.4122	0.5752	0.4094	0.9943	0.0123	0.5945		0.9130	0.9865	0.2084	0.3290	0.1998	0.9944	0.0156
QuadraticDiscriminantAnalysis	0.6142	0.7273	0.9943	0.5277	0.6826	0.5260	0.9955	0.0108	0.5899		1.0000	1.0000	0.0121	0.0003	0.0000	0.9884	0.0267
ExtraTreesClassifier	0.5937	0.6364	0.9965	0.6097	0.7496	0.6094	0.9949	0.0102	0.5280		0.5217	0.9952	0.5708	0.7162	0.5714	0.9899	0.0120

^a^ROC-AUC: area under the receiver operating characteristic curve.

^b^PPV: positive predictive value.

^c^NPV: negative predictive value.

^d^AUC: area under the curve.

^e^LGBMClassifier: light gradient boosting machine classifier.

^f^CatBoost: categorical boosting classifier.

^g^MLPClassifier: multilayer perceptron classifier.

^h^XGBClassifier: extreme gradient boosting classifier.

^i^NB: Naive Bayes.

### Model Interpretation

**Figure 3:**
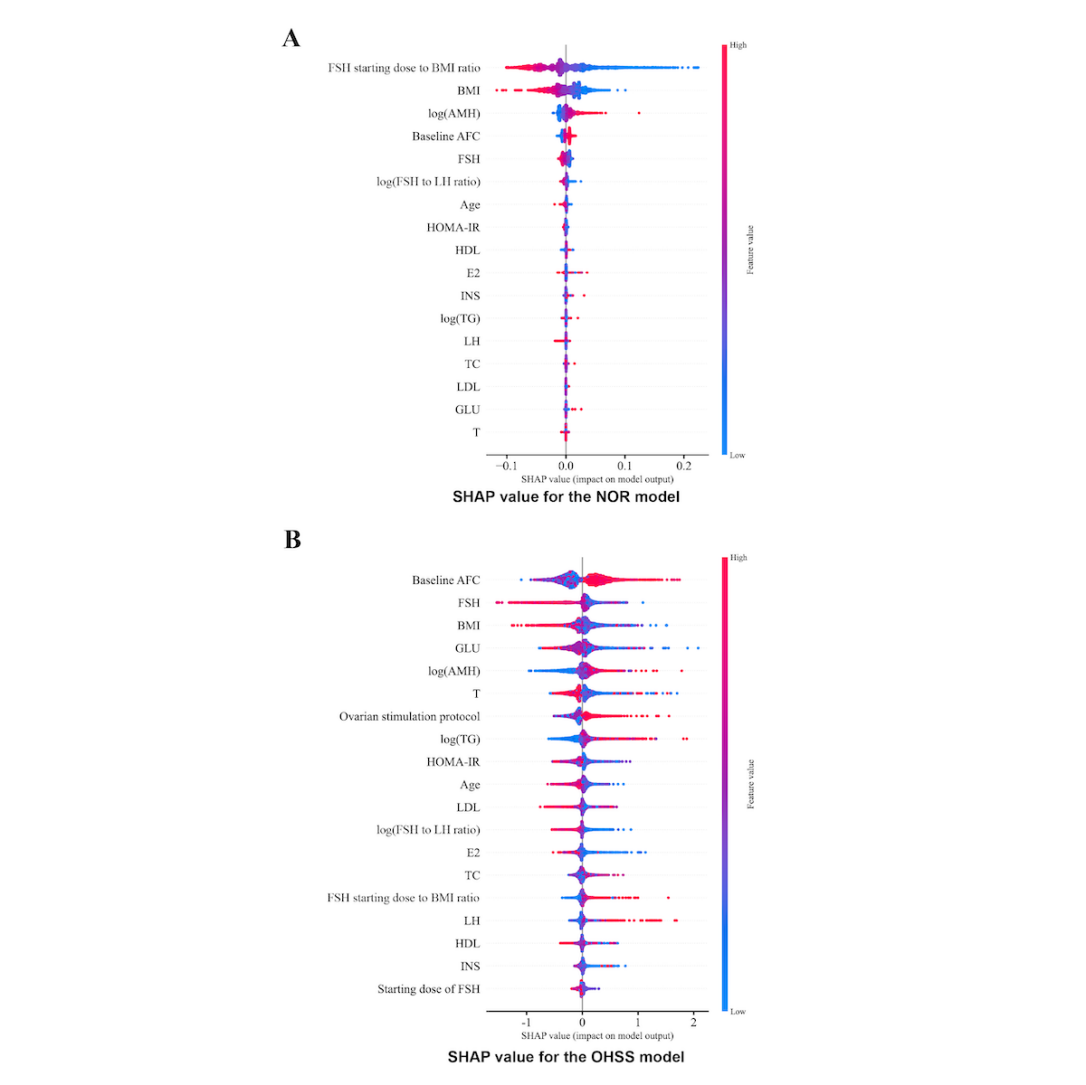
Shapley additive explanation (SHAP) values of the prediction models for (A) number of oocytes retrieved (NOR) and (B) ovarian hyperstimulation syndrome (OHSS). FSH: follicle-stimulating hormone; BMI, body mass index; AMH: anti-Müllerian hormone; AFC: antral follicle count; LH: luteinizing hormone; HOMA-IR: homeostatic model assessment of insulin resistance; HDL: high-density lipoprotein; E2: estradiol; INS: fasting insulin; TG: triglycerides; TC: total cholesterol; LDL: low-density lipoprotein; GLU: fasting glucose; T: testosterone;.

SHAP values were used to assess feature importance for both models, as shown in Figure 3. For NOR prediction, the features with the highest mean absolute SHAP values were FSH starting dose to BMI ratio, BMI, log (AMH), baseline AFC, and baseline FSH, indicating their significant contribution to the model. For early-onset moderate-to-severe OHSS prediction, the most important features identified were baseline AFC, followed by baseline FSH, BMI, fasting blood glucose, and log (AMH).

### Integrated Dose-Response Curves and Web Calculator

**Figure 4:**
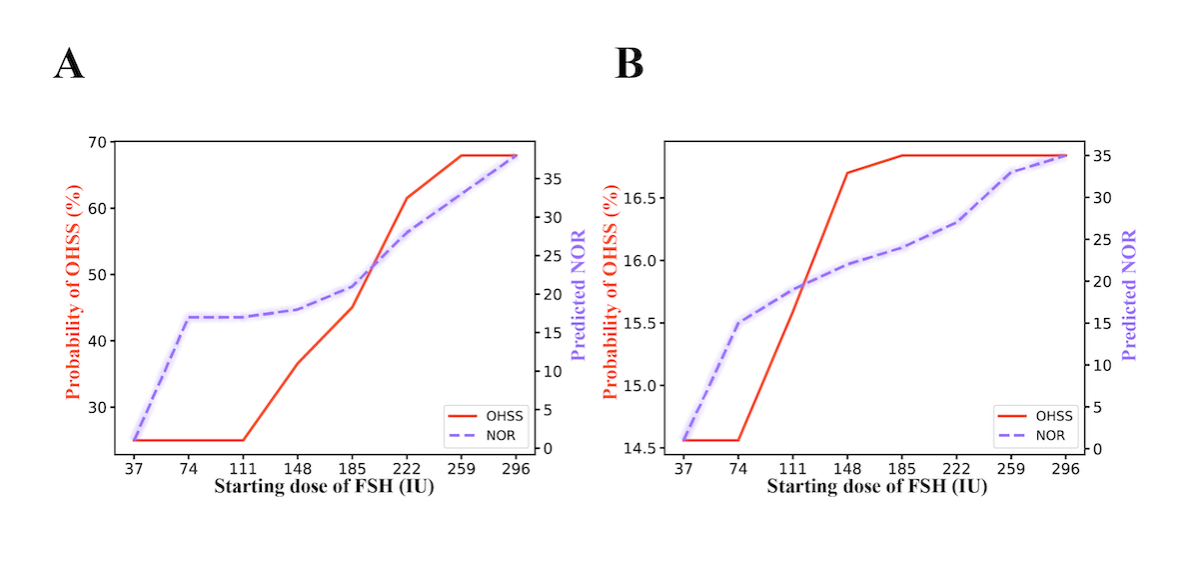
Model application and representative patient examples with predicted relatively high (A) and low (B) risks of ovarian hyperstimulation syndrome (OHSS). FSH: follicle-stimulating hormone; NOR: number of oocytes retrieved.

To facilitate individualized ovarian stimulation, we further integrated the prediction models for both NOR and early-onset moderate-to-severe OHSS into dose-response curves. Examples of patients with relatively high and low predicted risks of OHSS are presented in Figures 4A and 4B, respectively. As shown, increasing the starting dose of FSH leads to variable increases in both early-onset moderate-to-severe OHSS probability and predicted NOR for different patients. However, the probability of OHSS occurrence varies among individuals. On the basis of these personalized dose-response predictions, clinicians can determine a suitable starting dose of FSH to achieve an optimal NOR while maintaining a relatively low risk of early-onset moderate-to-severe OHSS.

Additionally, we developed a web-based calculator as a research-oriented prototype to enhance accessibility and facilitate exploratory use of the proposed models. The intuitive interface allows users to input relevant data and receive immediate predictions from the models (Figure S7 in Multimedia Appendix 1).

## Discussion

### Principal Findings

In this study, we developed an integrated ML-based system, “InOvaSGuide,” capable of simultaneously predicting NOR and the associated risk of early-onset moderate-to-severe OHSS across a wide range of FSH starting doses. By generating individualized dose-response curves, the model provides a continuous view of expected oocyte yield and corresponding safety profiles, providing clinicians with a structured visual reference to support individualized FSH dose selection. A web-based calculator was further implemented as a research-oriented prototype to improve accessibility and facilitate exploratory use of the models in clinically relevant scenarios.

Oocyte yield remains a key determinant of both efficacy and safety in assisted reproduction. Retrieval of fewer than 4 oocytes has been associated with poor reproductive prognosis [17], whereas obtaining more than 15 oocytes increase the likelihood of OHSS and may slightly compromise live birth outcomes [18,19]. Accordingly, a target range of 5 to 15 oocytes is generally recommended to balance benefit and risk [19]. The dose-response curve framework aligns conceptually with these clinical principles, as it illustrates how predicted NOR changes with increasing FSH doses, thereby supporting informed dosing discussions rather than prescriptive decision-making.

Existing ML-based NOR models generally focus either on approximating actual or optimal oocyte yield [11-13,20] or on producing individualized curves based on a limited number of clinical features [10]. In contrast, our approach used feature importance scores from automated classifiers for selection and compared 11 regression algorithms across 2 independent datasets. This allowed the construction of robust dose-response curves that illustrate how predicted NOR varies with incremental FSH doses, providing additional insight beyond traditional single-point estimates by visualizing predictions across a continuum of FSH doses.

We further developed ML models to predict OHSS, addressing a gap in existing clinical tools that predominantly rely on logistic regression [21,22] or receiver operating characteristic–based analyses [23,24]. Although contemporary strategies, including gonadotropin-releasing hormone antagonist protocols, dual triggering, and “freeze-all” approaches, have substantially reduced the incidence of early-onset OHSS, it remains a persistent concern even among presumed normal responders and has not been fully eliminated from clinical practice [25-27]. Our models demonstrated acceptable and consistent discriminatory ability across both internal and external cohorts, despite the limited number of OHSS events.

Importantly, the low prevalence of early-onset moderate-to-severe OHSS introduces substantial and unavoidable class imbalance, which has direct implications for model performance metrics. In particular, precision is structurally constrained in low-prevalence settings; therefore, PR-AUC values should be interpreted with caution. Although ROC-AUC indicated reasonable discrimination, PR-AUC is highly sensitive to outcome prevalence. When event rates fall below 1%, even well-calibrated models will inherently yield modest precision. In addition, our modeling strategy deliberately prioritized sensitivity to enhance clinical safety, an approach that increases false-positive predictions and further reduces precision and PR-AUC but minimizes the risk of missing true high-risk cases.

Within this context, the OHSS model should be viewed primarily as a screening and risk-stratification aid rather than a diagnostic or decision-making tool. Its intended role is to flag patients with potentially elevated risk who may warrant closer monitoring or consideration of preventive strategies, rather than to definitively predict OHSS occurrence or guide autonomous clinical actions.

Feature importance analyses largely reflected established biological associations. For NOR, the FSH starting dose to BMI ratio, BMI, and log (AMH) emerged as the most influential predictors. While most features were consistent with clinical practice, the contribution of metabolic markers, such as glucose, lipids, and metabolic indicators, warrants further investigation. For early-onset moderate-to-severe OHSS, AFC, baseline FSH, BMI, and log (AMH) emerged as dominant predictors, aligning with known determinants of ovarian reserve and ovarian sensitivity [23,28]. Associations involving testosterone or glucose were less pronounced, highlighting the multifactorial nature of OHSS risk, particularly in women with polycystic ovary syndrome [29]. Overall, these findings illustrate the capability of ML approaches to integrate diverse clinical variables and improve predictive performance.

A key practical advantage of this study is the integration of NOR and early-onset moderate-to-severe OHSS predictions into a unified, visually intuitive research-oriented system. InOvaSGuide enables clinicians to assess the potential trade-offs between stimulation efficacy and safety across a continuum of FSH doses. The web-based interface supports exploratory analysis and clinician-patient discussion; however, the system does not generate prescriptive dosing recommendations and is not intended for autonomous clinical use. Importantly, prospective validation is essential before any consideration of clinical deployment.

Beyond model performance, the development and potential deployment of AI-based, clinician-in-the-loop decision support tools in reproductive medicine entail careful ethical, legal, and implementation considerations [30,31]. Given the sensitivity of reproductive health data, rigorous safeguards for privacy protection and informed consent are essential [32]. Algorithmic transparency is equally important to support clinician interpretation and reduce risks of automation bias [33], while potential bias across patient subgroups remains an important consideration for future validation. In addition, AI-driven clinical tools may fall under medical software regulation, requiring evidence of safety and clinical validity before clinical implementation. Finally, effective integration into clinical practice will depend on usability, compatibility with established workflows, and clearly assigned clinical accountability [30]. Addressing these factors will be necessary before the system can be responsibly adopted in real-world settings.

### Limitations

Our study had several limitations. First, it focused on early-onset moderate-to-severe OHSS, excluding mild cases that may self-resolve and late-onset OHSS more commonly associated with embryo transfer. Patients with a predicted poor prognosis were also excluded under the assumption that they are less likely to develop OHSS. These exclusions introduced a structural selection bias that narrowed the population represented and limited the generalizability of the model in broader clinical settings. Second, the relatively small number of OHSS cases limited the model’s ability to fully characterize patients who are affected. This scarcity, together with the substantial class imbalance, also constrained the effectiveness of resampling-based strategies. Although multiple resampling methods were evaluated, only cost-sensitive learning may allow a more reliable assessment of alternative methods. Third, the retrospective nature of the study restricted the availability of certain relevant factors, such as previous OHSS history and genetic susceptibility, and may also introduce selection bias and unmeasured confounding that cannot be fully controlled. Finally, although the system provides individualized dose-response curves for clinical reference, it does not generate a prescriptive starting dose. Moreover, the model has not yet undergone prospective evaluation, which limits its current clinical applicability. A prospective validation study is planned as a necessary next step to assess real-world performance. Future large-scale, multicenter validation in broader patient populations will be essential for improving model stability and generalizability.

### Conclusions

We developed and externally validated InOvaSGuide, a ML system that simultaneously predicts NOR and early-onset moderate-to-severe OHSS risk across a continuum of FSH doses. By linking efficacy and safety within a single dose-response framework, the tool highlights the broader potential of model-informed dosing to standardize ovarian stimulation and enhance patient safety. Prospective trials are needed to establish real-world utility.

## Appendix

Multimedia Appendix 1 Data supporting model development and validation, including the distribution and transformation of key variables, feature selection for number of oocytes retrieved and ovarian hyperstimulation syndrome prediction, confusion matrices, the web-based calculator interface, and detailed patient baseline characteristics, together with the completed TRIPOD+AI checklist.

## Abbreviations

AFC: antral follicle count
AI: artificial intelligence
AMH: anti-Müllerian hormone
ROC-AUC: area under the receiver operating characteristic curve
FSH: follicle-stimulating hormone
InOvaSGuide: individualized ovarian stimulation guide
IVF: in vitro fertilization
LH: luteinizing hormone
NOR: number of oocytes retrieved
OHSS: ovarian hyperstimulation syndrome
PR-AUC: precision-recall area under the curve
SHAP: Shapley additive explanation
TRIPOD: Transparent Reporting of a Multivariable Prediction Model for Individual Prognosis or Diagnosis

## Data Availability

The datasets generated or analyzed during this study are available from the corresponding author on reasonable request.
