# Supplementary material for: Integrated Prediction System for Individualized Ovarian Stimulation and Ovarian Hyperstimulation Syndrome Prevention: Algorithm Development and Validation
Source: J Med Internet Res. 2026 Feb 3;28:e78245. doi: 10.2196/78245 (PMC12914236; doi:10.2196/78245)
Supplement: Multimedia Appendix 1 [file jmir_v28i1e78245_app1.pdf]

Supplementary Material

Table of contents

Material	Content	Page
Figure S1	Frequency distribution histograms of AMH, TG, FSH/LH levels, and the outcome variables before and after log transform	2
Figure S2	Scatter plot of FSH/LH, AMH, TG before and after log transformation	3
Figure S3	Feature selection based on the Random Forest Regression model for NOR prediction	4
Figure S4	Feature selection based on the LightGBM model for OHSS prediction	5
Figure S5	Confusion matrix in the internal dataset	6
Figure S6	Confusion matrix in the external dataset	6
Figure S7	The interactive interface of the web calculator within the InOvaSGuide system	7
Table S1	TRIPOD+AI checklist	8-9
Table S2	Detailed model information for NOR prediction	10
Table S3	Detailed model information for OHSS prediction	10
Table S4	Baseline characteristics of patients with and without OHSS in the internal dataset	10-11
Table S5	Baseline characteristics of patients with and without OHSS in the external dataset	12

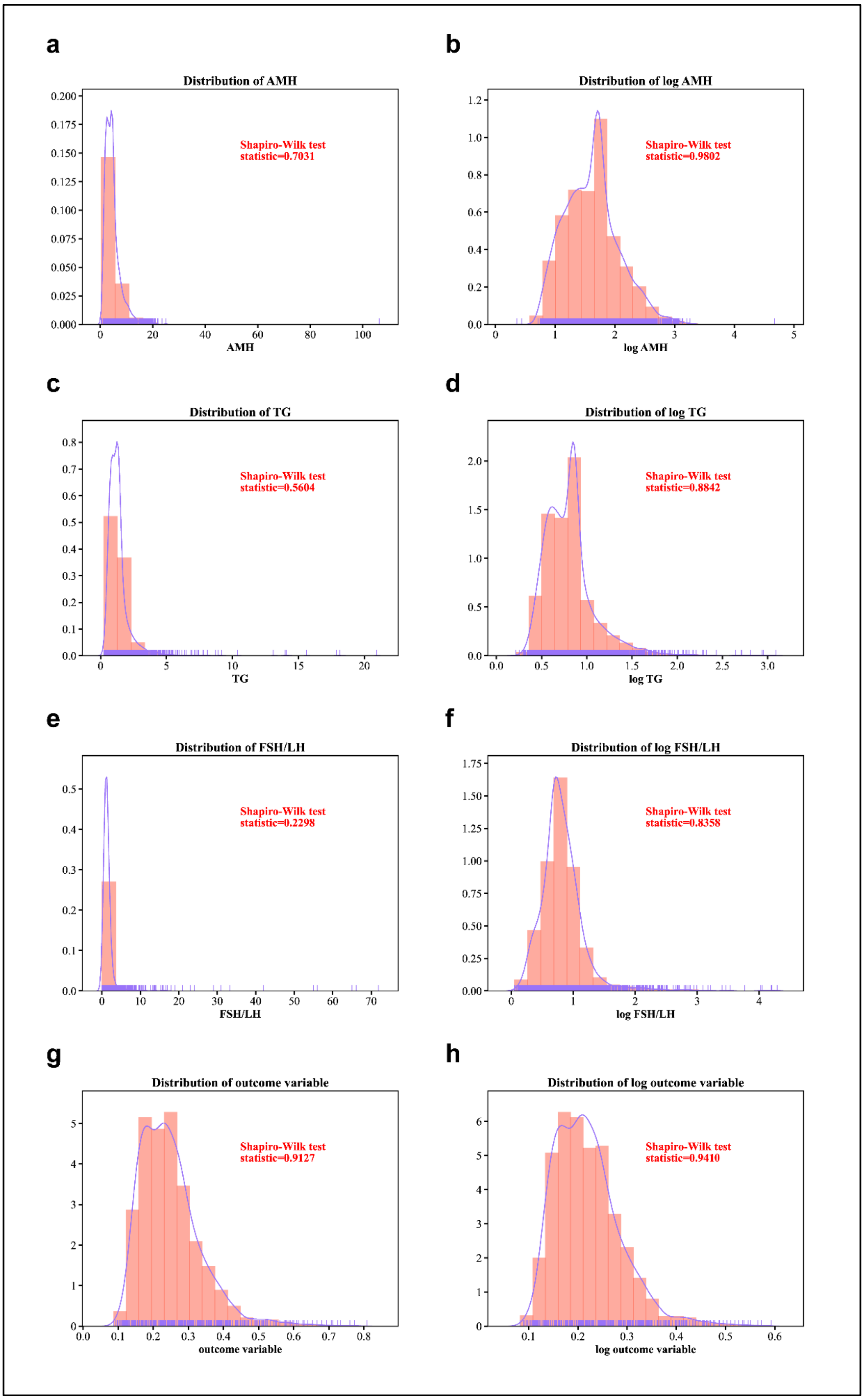

Figure S1. Frequency distribution histograms of AMH, TG, FSH/LH levels, and the outcome variables before and after log transform. AMH, anti-Müllerian hormone; TG, triglycerides; FSH, follicle-stimulating hormone; LH, luteinizing hormone.

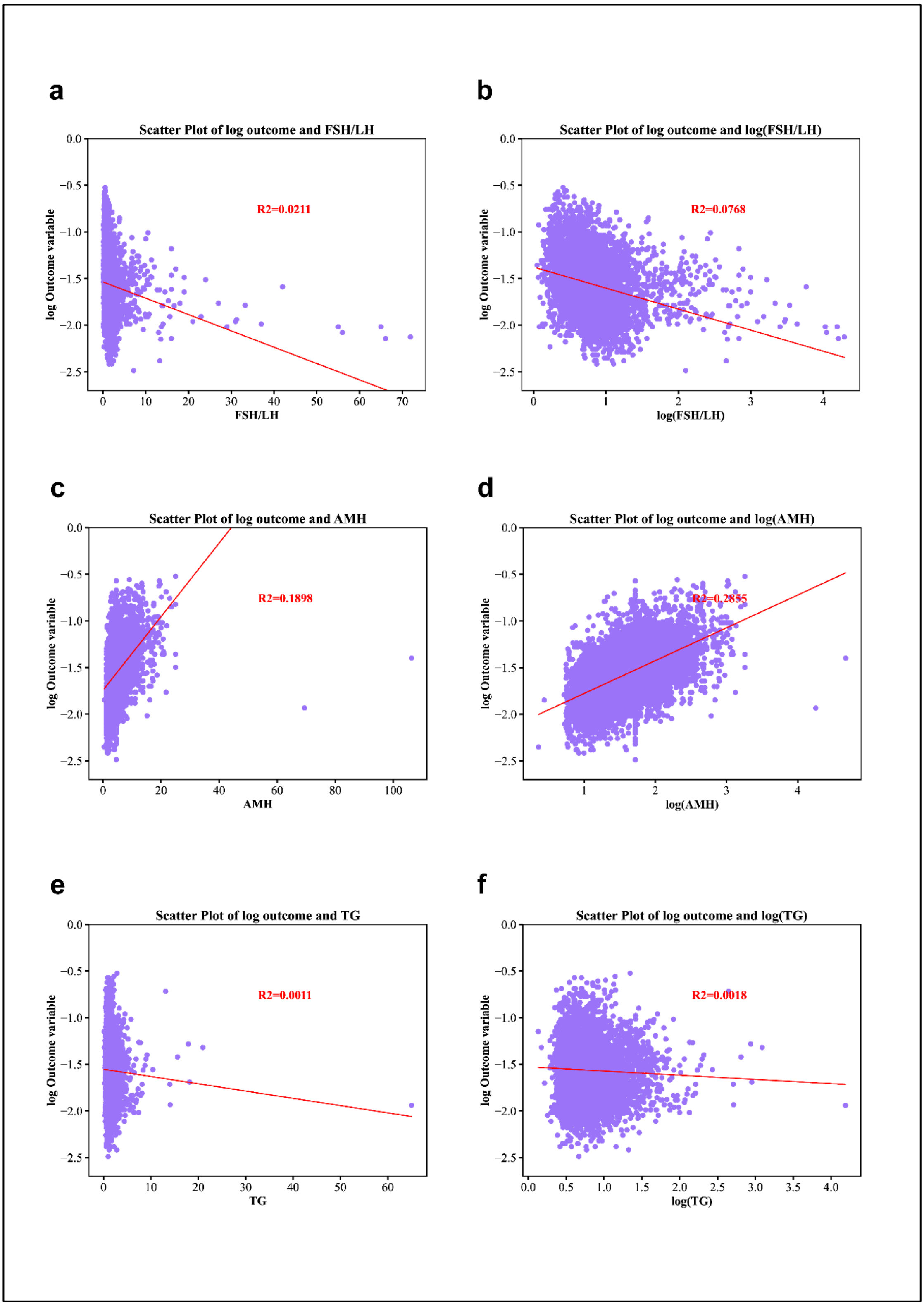

Figure S2. Scatter plot of FSH/LH, AMH, TG before and after log transformation. (a, b) FSH/LH. (c, d) AMH. (e, f) TG. AMH, anti-Müllerian hormone; TG, triglycerides; FSH, follicle-stimulating hormone; LH, luteinizing hormone.

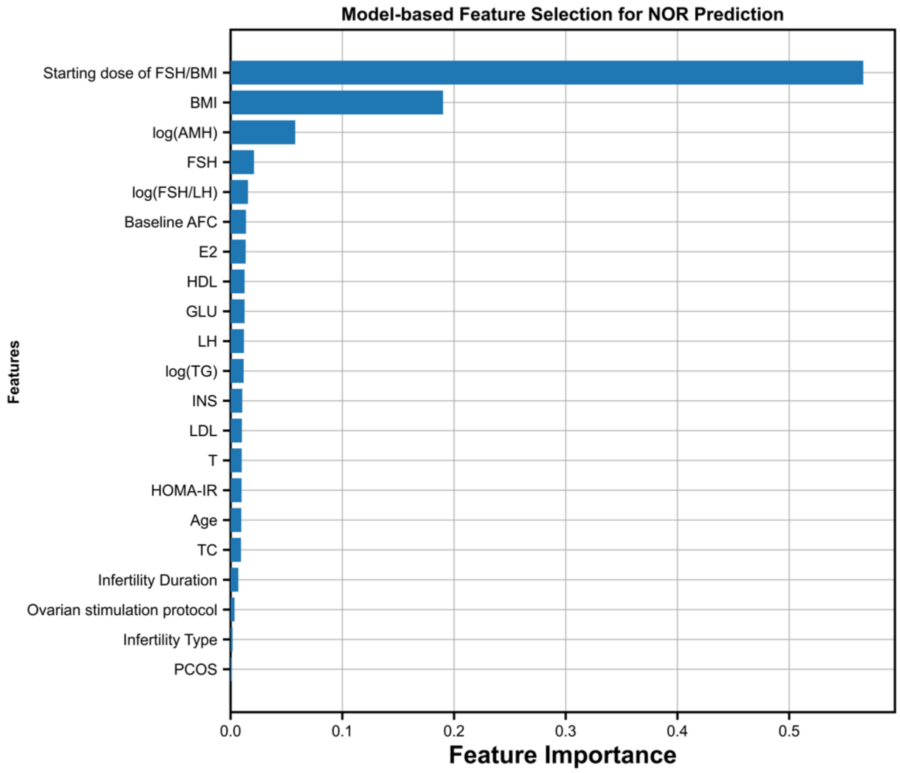

Figure S3. Feature selection based on the Random Forest Regression model for NOR prediction. NOR, number of oocytes retrieved; FSH, follicle-stimulating hormone; BMI: body mass index; AMH, anti-Müllerian hormone; LH, luteinizing hormone; AFC, antral follicle count; E2, estradiol; HDL, high-density lipoprotein; GLU, fasting glucose; TG, triglycerides; INS, fasting insulin; LDL, low-density lipoprotein; T, testosterone; HOMA-IR, homeostatic model assessment of insulin resistance; TC, total cholesterol; PCOS, polycystic ovarian syndrome.

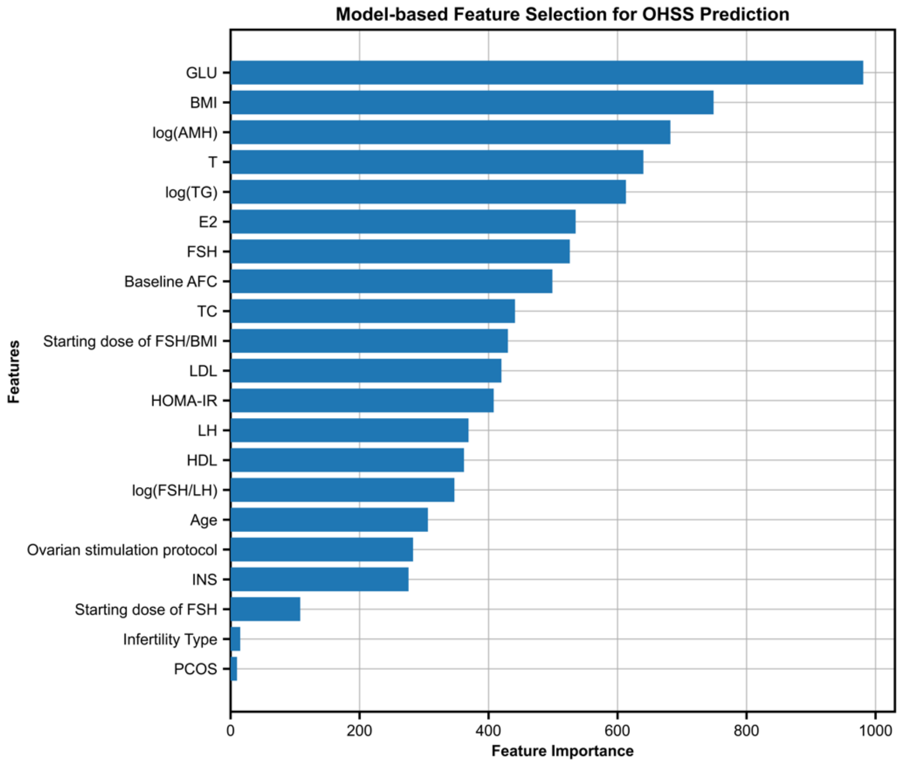

Figure S4. Feature selection based on the LightGBM model for OHSS prediction. LightGBM, light Gradient boosting machine classifier; OHSS, ovarian hyperstimulation syndrome; GLU, fasting glucose; BMI: body mass index; AMH, anti-Müllerian hormone; T, testosterone; TG, triglycerides; E2: estradiol; FSH, follicle-stimulating hormone; AFC, antral follicle count; TC, total cholesterol; LDL, low-density lipoprotein; HDL, high-density lipoprotein; HOMA-IR， homeostasis model assessment of insulin resistance； LH, luteinizing hormone; INS, fasting insulin; PCOS, polycystic ovarian syndrome.

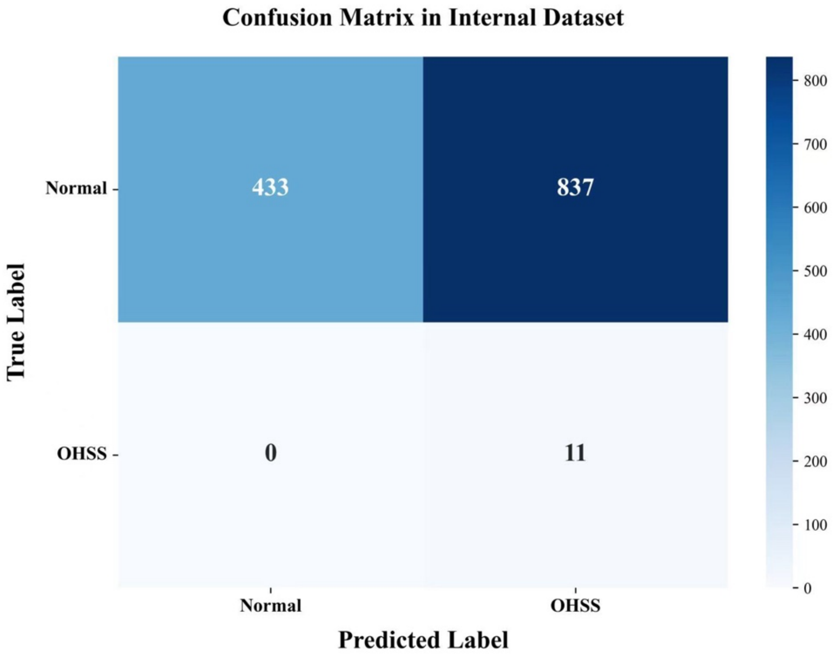

Figure S5. Confusion matrix in the internal dataset. OHSS, ovarian hyperstimulation syndrome;

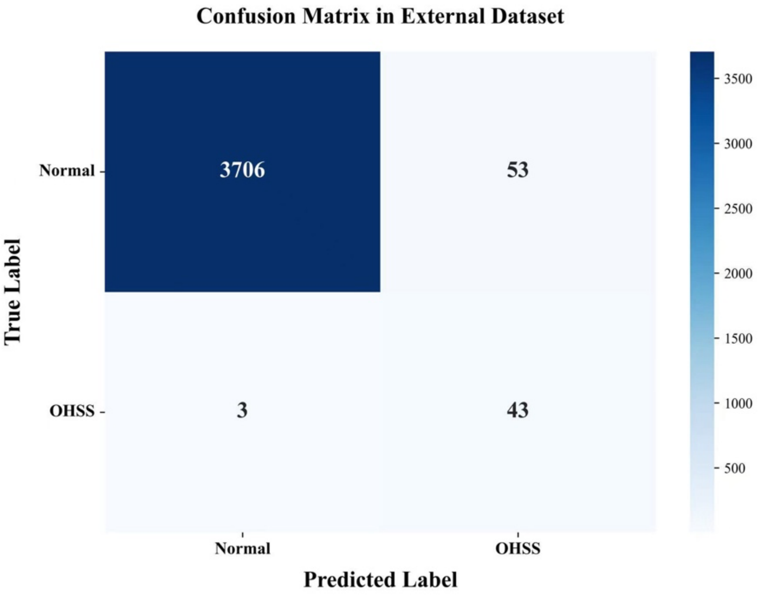

Figure S6. Confusion matrix in the external dataset. OHSS, ovarian hyperstimulation syndrome;

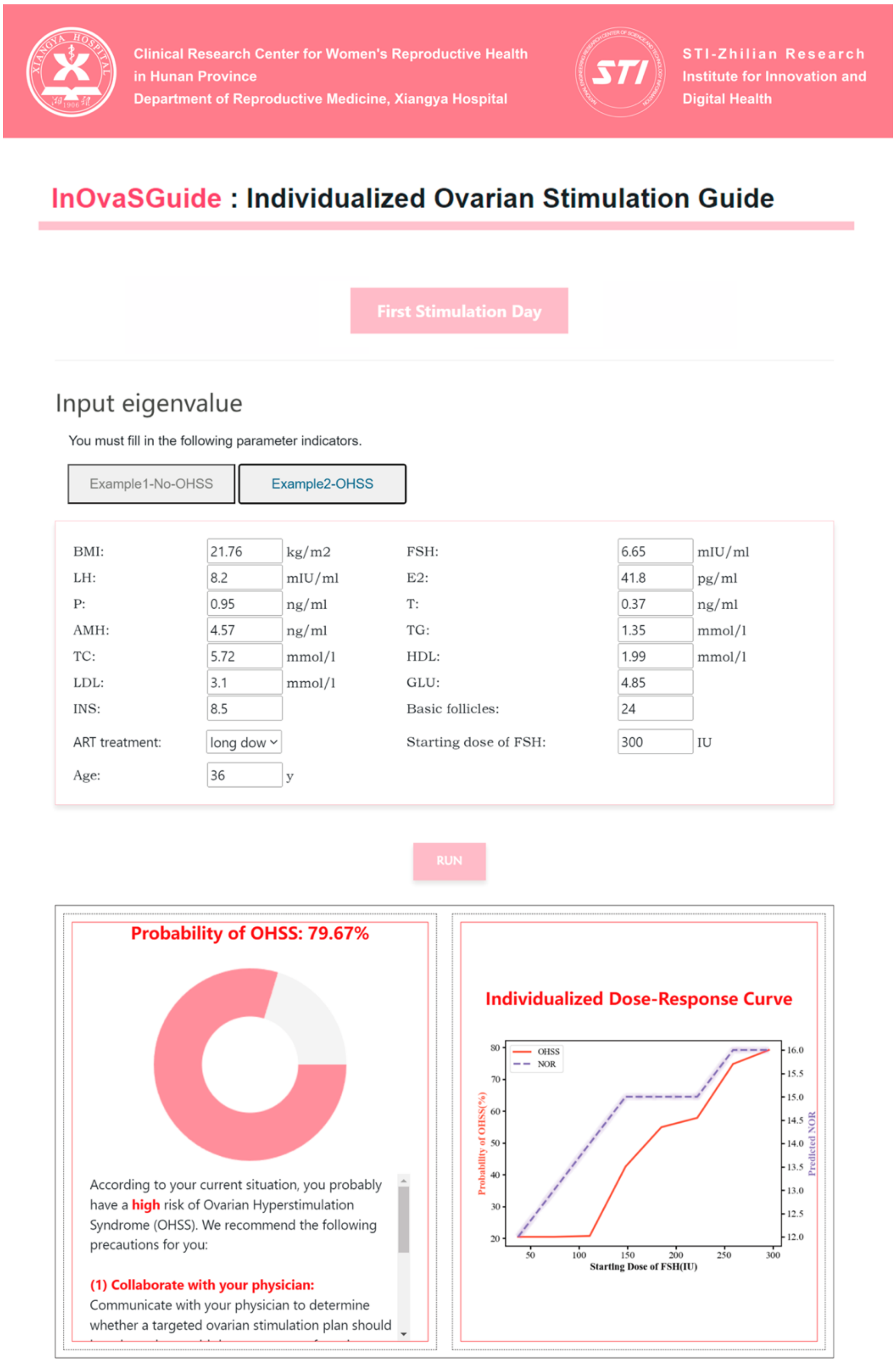

Figure S7. The interactive interface of the web calculator within the InOvaSGuide system. Abbreviation: OHSS, ovarian hyperstimulation syndrome; NOR, number of oocytes retrieved; AFC, antral follicle count; FSH, follicle-stimulating hormone; BMI: body mass index; GLU, fasting glucose; AMH, anti-Müllerian hormone; T, testosterone; TG, triglycerides; HOMA-IR, homeostatic model assessment of insulin resistance; LDL, low-density lipoprotein; E2, estradiol; TC, total cholesterol; LH, luteinizing hormone; HDL, high-density lipoprotein; INS, fasting insulin; Gn, gonadotrophin.

Section/Topic	Item	Development / evaluation[1]	Checklist item	Reported on page
**TITLE**				
Title	1	D;E	Identify the study as developing or evaluating the performance of a multivariable prediction model, the target population, and the outcome to be predicted	1
**ABSTRACT**				
Abstract	2	D;E	See TRIPOD+AI for Abstracts checklist	1
**INTRODUCTION**				
Background	3a	D;E	Explain the healthcare context (including whether diagnostic or prognostic) and rationale for developing or evaluating the prediction model, including references to existing models	2-3
	3b	D;E	Describe the target population and the intended purpose of the prediction model in the context of the care pathway, including its intended users (e.g., healthcare professionals, patients, public)	2-3
	3c	D;E	Describe any known health inequalities between sociodemographic groups	2
Objectives	4	D;E	Specify the study objectives, including whether the study describes the development or validation of a prediction model (or both)	3
**METHODS**				
Data	5a	D;E	Describe the sources of data separately for the development and evaluation datasets (e.g., randomised trial, cohort, routine care or registry data), the rationale for using these data, and representativeness of the data	3-4
	5b	D;E	Specify the dates of the collected participant data, including start and end of participant accrual; and, if applicable, end of follow-up	3-4
Participants	6a	D;E	Specify key elements of the study setting (e.g., primary care, secondary care, general population) including the number and location of centres	3-4
	6b	D;E	Describe the eligibility criteria for study participants	3-4
	6c	D;E	Give details of any treatments received, and how they were handled during model development or evaluation, if relevant	4-5
Data preparation	7	D;E	Describe any data pre-processing and quality checking, including whether this was similar across relevant sociodemographic groups	5
Outcome	8a	D;E	Clearly define the outcome that is being predicted and the time horizon, including how and when assessed, the rationale for choosing this outcome, and whether the method of outcome assessment is consistent across sociodemographic groups	5-6
	8b	D;E	If outcome assessment requires subjective interpretation, describe the qualifications and demographic characteristics of the outcome assessors	NA
	8c	D;E	Report any actions to blind assessment of the outcome to be predicted	3
Predictors	9a	D	Describe the choice of initial predictors (e.g., literature, previous models, all available predictors) and any pre-selection of predictors before model building	5
	9b	D;E	Clearly define all predictors, including how and when they were measured (and any actions to blind assessment of predictors for the outcome and other predictors)	4
	9c	D;E	If predictor measurement requires subjective interpretation, describe the qualifications and demographic characteristics of the predictor assessors	NA
Sample size	10	D;E	Explain how the study size was arrived at (separately for development and evaluation), and justify that the study size was sufficient to answer the research question. Include details of any sample size calculation	5
Missing data	11	D;E	Describe how missing data were handled. Provide reasons for omitting any data	5
Analytical methods	12a	D	Describe how the data were used (e.g., for development and evaluation of model performance) in the analysis, including whether the data were partitioned, considering any sample size requirements	5-8
	12b	D	Depending on the type of model, describe how predictors were handled in the analyses (functional form, rescaling, transformation, or any standardisation).	5
	12c	D	Specify the type of model, rationale[2], all model-building steps, including any hyperparameter tuning, and method for internal validation	6
	12d	D;E	Describe if and how any heterogeneity in estimates of model parameter values and model performance was handled and quantified across clusters (e.g., hospitals, countries). See TRIPOD-Cluster for additional considerations[3]	NA
	12e	D;E	Specify all measures and plots used (and their rationale) to evaluate model performance (e.g., discrimination, calibration, clinical utility) and, if relevant, to compare multiple models	5-11,14
	12f	E	Describe any model updating (e.g., recalibration) arising from the model evaluation, either overall or for particular sociodemographic groups or settings	NA
	12g	E	For model evaluation, describe how the model predictions were calculated (e.g., formula, code, object, application programming interface)	14
Class imbalance	13	D;E	If class imbalance methods were used, state why and how this was done, and any subsequent methods to recalibrate the model or the model predictions	7-8
Fairness	14	D;E	Describe any approaches that were used to address model fairness and their rationale	NA
Model output	15	D	Specify the output of the prediction model (e.g., probabilities, classification). Provide details and rationale for any classification and how the thresholds were identified	5

[1] D=items relevant only to the development of a prediction model; E=items relating solely to the evaluation of a prediction model; D;E=items applicable to both the development and evaluation of a prediction model
[2] Separately for all model building approaches.
[3] TRIPOD-Cluster is a checklist of reporting recommendations for studies developing or validating models that explicitly account for clustering or explore heterogeneity in model performance (eg, at different hospitals or centres). Debray et al, BMJ 2023; 380: e071018 [DOI: 10.1136/bmj-2022-071018]

Training versus evaluation	16	D;E	Identify any differences between the development and evaluation data in healthcare setting, eligibility criteria, outcome, and predictors	Table 1
Ethical approval	17	D;E	Name the institutional research board or ethics committee that approved the study and describe the participant-informed consent or the ethics committee waiver of informed consent	3
**OPEN SCIENCE**				
Funding	18a	D;E	Give the source of funding and the role of the funders for the present study	19
Conflicts of interest	18b	D;E	Declare any conflicts of interest and financial disclosures for all authors	20
Protocol	18c	D;E	Indicate where the study protocol can be accessed or state that a protocol was not prepared	NA
Registration	18d	D;E	Provide registration information for the study, including register name and registration number, or state that the study was not registered	NA
Data sharing	18e	D;E	Provide details of the availability of the study data	19
Code sharing	18f	D;E	Provide details of the availability of the analytical code[4]	NA
**PATIENT & PUBLIC INVOLVEMENT**				
Patient & Public Involvement	19	D;E	Provide details of any patient and public involvement during the design, conduct, reporting, interpretation, or dissemination of the study or state no involvement.	NA
**RESULTS**				
Participants	20a	D;E	Describe the flow of participants through the study, including the number of participants with and without the outcome and, if applicable, a summary of the follow-up time. A diagram may be helpful.	NA
	20b	D;E	Report the characteristics overall and, where applicable, for each data source or setting, including the key dates, key predictors (including demographics), treatments received, sample size, number of outcome events, follow-up time, and amount of missing data. A table may be helpful. Report any differences across key demographic groups.	Table 1
	20c	E	For model evaluation, show a comparison with the development data of the distribution of important predictors (demographics, predictors, and outcome).	Table 1, S3, S4
Model development	21	D;E	Specify the number of participants and outcome events in each analysis (e.g., for model development, hyperparameter tuning, model evaluation)	6
Model specification	22	D	Provide details of the full prediction model (e.g., formula, code, object, application programming interface) to allow predictions in new individuals and to enable third-party evaluation and implementation, including any restrictions to access or re-use (e.g., freely available, proprietary)[5]	NA
Model performance	23a	D;E	Report model performance estimates with confidence intervals, including for any key subgroups (e.g., sociodemographic). Consider plots to aid presentation.	NA
	23b	D;E	If examined, report results of any heterogeneity in model performance across clusters. See TRIPOD Cluster for additional details[3].	NA
Model updating	24	E	Report the results from any model updating, including the updated model and subsequent performance	NA
**DISCUSSION**				
Interpretation	25	D;E	Give an overall interpretation of the main results, including issues of fairness in the context of the objectives and previous studies	19
Limitations	26	D;E	Discuss any limitations of the study (such as a non-representative sample, sample size, overfitting, missing data) and their effects on any biases, statistical uncertainty, and generalizability	18
Usability of the model in the context of current care	27a	D	Describe how poor quality or unavailable input data (e.g., predictor values) should be assessed and handled when implementing the prediction model	5
	27b	D	Specify whether users will be required to interact in the handling of the input data or use of the model, and what level of expertise is required of users	NA
	27c	D;E	Discuss any next steps for future research, with a specific view to applicability and generalizability of the model	18

From: Collins GS, Moons KGM, Dhiman P, et al. *BMJ* 2024;385:e078378. doi:10.1136/bmj-2023-078378

[4] This relates to the analysis code, for example, any data cleaning, feature engineering, model building, evaluation.
[5] This relates to the code to implement the model to get estimates of risk for a new individual.

Table S2. Detailed model information for NOR prediction

ML algorithms	Hyperparameter
Linear Regression	Linear Regression ()
XGB Regressor	XGB Regressor (n_estimators=300, learning_rate=0.01, random_state=999)
LGBM Regressor	LGBM Regressor (n_estimators=300, learning_rate=0.01, random_state=999)
Random Forest Regressor	Random Forest Regressor (n_estimators=300, random_state=999)
SVR	SVR (kernel='rbf')
Gradient Boosting Regressor	Gradient Boosting Regressor (random_state=999)
Ridge	Ridge (random_state=999, alpha=1.0)
Lasso	Lasso (alpha=1.0)
Elastic Net	Elastic Net (alpha=1.0, l1_ratio=0.5)
Decision Tree Regressor	Decision Tree Regressor ()
MLP Regressor	MLP Classifier (random_state=999)

Abbreviation: NOR, number of oocytes retrieved; ML, model learning; XGB Regressor, extreme gradient boosting regressor; LGBM Regressor, light gradient boosting machine regressor; SVR, support vector regression; MLP, multi-layer perceptron.

Table S3. Detailed model information for OHSS prediction

ML algorithms	Hyperparameter
Extra-trees	Extra Trees Classifier (random_state=999, class_weight="balanced")
Gradient boosting machine	Gradient Boosting Classifier (random_state=999)
Cat Boost	Cat Boost Classifier (random_state=999, verbose=0)
Gaussian naive Bayes (NB)	Gaussian NB ()
Linear discriminant analysis	Linear Discriminant Analysis ()
Quadratic discriminant analysis	Quadratic Discriminant Analysis ()
Multi-layer perceptron	MLP Classifier (random_state=999)
Logistic regression	Logistic Regression (random_state=999)
Random forest	Random Forest Classifier (n_estimators=350, random_state=42, class_weight='balanced')
XGB Classifier	XGB Classifier (n_estimators=300, scale_pos_weight=150, learning_rate=0.05)
LGBM Classifier	LGBM Classifier (n_estimators=300, random_state=42, sample_weight=500, learning_rate=0.01)

Abbreviation: OHSS, ovarian hyperstimulation syndrome; ML, model learning; Ada boost, adaptive boost classifier; Cat Boost, categorical boosting classifier; MLP, multi-layer perceptron classifier; XGB, extreme gradient boosting classifier; LGBM Classifier, light gradient boosting machine classifier.

Table S4. Baseline characteristics of patients with and without OHSS in the internal dataset

	non-OHSS (n=6346)	OHSS (n=55)	P value
Age, mean (SD), y	30.0 (3.85)	28.7 (3.00)	0.002
BMI, mean (SD), kg/m^2	22.1 (3.39)	21.4 (2.43)	0.041

	non-OHSS (n=6346)	OHSS (n=55)	P value
Baseline FSH, mean (SD), mIU/ml	6.25 (1.87)	5.52 (1.34)	<0.001
Baseline LH, mean (SD), mIU/ml	6.16 (4.56)	7.66 (5.53)	0.051
AMH, mean (SD), ng/ml	4.56 (3.25)	6.72 (4.00)	<0.001
PCOS, n (%)			
non-PCOS	5414 (85.3)	41 (74.5)	0.040
PCOS	932 (14.7)	14 (25.5)	
Infertility duration, mean (SD), y	3.82 (2.71)	3.58 (2.09)	0.386
Infertility type, n (%)			
primary infertility	3332 (52.5)	34 (61.8)	0.214
secondary infertility	3014 (47.5)	21 (38.2)	
Etiology of infertility, n (%)			
Tubal	5243 (82.6)	43 (78.2)	0.388
Male factors	612 (9.6)	6 (10.9)	
RSA	276 (4.3)	2 (3.6)	
Others	209 (3.3)	4 (7.3)	
Fasting blood glucose, mean (SD), mmol/L	5.30 (0.52)	5.18 (0.35)	0.017
Fasting insulin, mean (SD), uU/ml	11.0 (5.69)	10.2 (5.71)	0.300
HOMA-IR, mean (SD)	2.63 (1.46)	2.35 (1.29)	0.124
Baseline AFC, mean (SD), n	19.0 (6.26)	22.7 (5.35)	<0.001
Ovarian stimulation protocol, n (%)			
GnRH agonist long protocol	1206 (19.0)	5 (9.1)	0.036
GnRH antagonist protocol	2631 (41.5)	19 (34.5)	
Early-follicular phase long-acting GnRH agonist long protocol	2273 (35.8)	27 (49.1)	
Ultra-long GnRH agonist protocol	236 (3.7)	4 (7.3)	
Starting dose of FSH, mean (SD), IU	170 (43.0)	153 (34.2)	0.001
Total dose of FSH, mean (SD), IU	2034 (729)	1839 (555)	0.012
Duration of stimulation, mean (SD), d	10.7 (2.4)	10.8 (2.2)	0.711
E2 level on the triggering day, mean (SD), pg/ml	3259 (1487)	3902 (1361)	0.001
Type of trigger, n (%)			
hCG	5512 (86.9)	52 (94.5)	0.322
hCG + GnRH-agonist	453 (7.1)	2 (3.6)	
GnRH-agonist	381 (6.0)	1 (1.8)	
Number of Follicles ≥14mm, mean (SD), n	3.79 (1.58)	4.24 (1.77)	0.068
Oocytes retrieved, mean (SD), n	13.9 (6.57)	23.9 (8.55)	<0.001
OHSS degree, n (%)	13.0 (3.71)	15.2 (4.05)	
Normal	6346 (100)	0 (0.00)	<0.001
Moderate	0 (0.00)	54 (98.2)	
Severe	0 (0.00)	1 (1.82)	
Num. of cycles with embryo transfer, mean (SD), n (%)	4053 (63.9)	10 (18.2)	<0.001
Num. of transferred embryos, mean (SD), n	1.75 (0.43)	1.40 (0.52)	0.061

	non-OHSS (n=6346)	OHSS (n=55)	P value
Ectopic pregnancy rate, n (%)	57 (1.41)	0 (0.00)	1.000
Intrauterine pregnancy rate, n (%)	2094 (51.7)	6 (60.0)	0.755
Miscarriage rate, n (%)	267/2094 (12.8)	1/6 (16.7)	0.560
Live birth, n (%)	1818/4053 (44.9)	5/10 (50.0)	0.761
Loss to follow-up, n (%)	9/4053 (0.2)	0/10 (0.0)	1.00

Abbreviation: SD, standard deviation; BMI: body mass index; FSH: follicle-stimulating hormone; LH: luteinizing hormone; AMH, anti-Müllerian hormone; HOMA-IR: homeostasis model assessment of insulin resistance; AFC: antral follicle count; GnRH: gonadotropin-releasing hormone; hCG: human chorionic gonadotropin; E2: estradiol; OHSS: ovarian hyperstimulation syndrome; PCOS, polycystic ovarian syndrome; RSA, recurrent spontaneous abortion.

Table S5. Baseline characteristics of patients with and without OHSS in the external dataset

	non-OHSS (n=3759)	OHSS (n=46)	P value
Age, mean (SD), y	31.8 (3.93)	31.0 (3.28)	0.086
BMI, mean (SD), kg/m^2	21.9 (3.03)	22.3 (3.67)	0.575
Baseline FSH, mean (SD), mIU/ml	6.61 (1.82)	6.21 (1.61)	0.107
Baseline LH, mean (SD), mIU/ml	5.89 (3.68)	6.01 (2.86)	0.772
AMH, mean (SD), ng/ml	4.52 (2.93)	6.04 (3.19)	0.002
Fasting blood glucose, mean (SD), mmol/L	4.60 (0.69)	4.77 (1.00)	0.249
Fasting insulin, mean (SD), uU/ml	62.7 (33.9)	58.5 (27.6)	0.316
HOMA-IR, mean (SD)	13.3 (7.60)	12.6 (5.20)	0.403
Baseline AFC, mean (SD), n	14.8 (6.60)	17.9 (8.42)	0.017
Ovarian stimulation protocol, n (%)			
GnRH agonist long protocol	1793 (47.7)	34 (73.9)	0.003
GnRH-antagonist protocol	1901 (50.6)	12 (26.1)	
ultra-long GnRH-agonist protocol	65 (1.7)	0 (0.0)	
Starting dose of FSH, mean (SD), IU	214 (62.1)	198 (59.1)	0.087
Total dose of FSH, mean (SD), IU	2230 (825)	2083 (763)	0.203
E2 level on the triggering day, mean (SD), pg/ml	3194 (2081)	4041 (2001)	0.006
Number of follicles ≥14mm, mean (SD), n	12.4 (5.09)	16.3 (5.67)	<0.001
Oocytes retrieved, mean (SD), n	15.4 (7.63)	23.1 (11.0)	<0.001

Abbreviation: SD, standard deviation; BMI: body mass index; FSH: follicle-stimulating hormone; LH: luteinizing hormone; AMH, anti-Müllerian hormone; HOMA-IR: homeostasis model assessment of insulin resistance; AFC: antral follicle count; GnRH: gonadotropin-releasing hormone; hCG: human chorionic gonadotropin; E2: estradiol; OHSS: ovarian hyperstimulation syndrome.
